# A platform utilizing *Drosophila* ovulation for nonhormonal contraceptive screening

**DOI:** 10.1073/pnas.2026403118

**Published:** 2021-07-06

**Authors:** Kewa Jiang, Jiyang Zhang, Yuping Huang, Yingzheng Wang, Shuo Xiao, M. Kyle Hadden, Teresa K. Woodruff, Jianjun Sun

**Affiliations:** ^a^Department of Physiology and Neurobiology, University of Connecticut, Storrs, CT 06269;; ^b^Department of Obstetrics and Gynecology, Northwestern University, Chicago, IL 60611;; ^c^Department of Pharmacology and Toxicology, Ernest Mario School of Pharmacy, Environmental and Occupational Health Sciences Institute, Rutgers University, Piscataway, NJ 08854;; ^d^Department of Pharmaceutical Sciences, School of Pharmacy, University of Connecticut, Storrs, CT 06269;; ^e^Institute for Systems Genomics, University of Connecticut, Storrs, CT 06269

**Keywords:** contraceptive screening, follicle rupture, nonsteroidal contraceptives, chlorpromazine

## Abstract

A significant unmet need for new contraceptive options for both women and men remains due to side-effect profiles, medical concerns, and the inconvenience of many currently available contraceptive products. Unfortunately, the development of novel nonsteroidal female contraceptive medicine has been stalled in the last couple of decades due to the lack of effective screening platforms. *Drosophila* utilizes conserved signaling pathways for follicle rupture, a final step in ovulation that is essential for female reproduction. Therefore, we explored the potential to use *Drosophila* as a model to screen compounds that could inhibit follicle rupture and be nonsteroidal contraceptive candidates. Using our ex vivo follicle rupture assay, we screened 1,172 Food and Drug Administration (FDA)–approved drugs and identified six drugs that could inhibit *Drosophila* follicle rupture in a dose-dependent manner. In addition, we characterized the molecular actions of these drugs in the inhibition of adrenergic signaling and follicle rupture. Furthermore, we validated that three of the four drugs consistently inhibited mouse follicle rupture in vitro and that two of them did not affect progesterone production. Finally, we showed that chlorpromazine, one of the candidate drugs, can significantly inhibit mouse follicle rupture in vivo. Our work suggests that *Drosophila* ovulation is a valuable platform for identifying lead compounds for nonsteroidal contraceptive development and highlights the potential of these FDA-approved drugs as novel nonsteroidal contraceptive agents.

## Significance

Due to undesirable side effects and other risk factors of current hormonal contraceptives, the world has increasing demand for novel nonsteroidal contraceptives. Unfortunately, the development of nonsteroidal contraceptives for women is completely stalled due to little investment from big pharmaceutical companies in this field and lack of effective screening platforms. In this manuscript, we describe a phenotypic screening platform utilizing a *Drosophila* ovulation assay to identify lead compounds that can efficiently inhibit follicle rupture, a final step of releasing mature oocytes during ovulation. In addition, we demonstrate that lead compounds identified from *Drosophila* ovulation could inhibit follicle rupture in mice and have great potential to become nonsteroidal contraceptives for women.

Hormonal contraceptive methods are the most common form of birth control among women of childbearing age globally. However, undesirable side effects, risk factors, and contraindications of hormonal contraceptives frequently lead to discontinuation, which ultimately results in increased rates of unintended pregnancies and abortions (1–3). Only 14% of women aged 15 to 49 use oral contraception in the United States, according to a recent National Center for Health Statistics data brief (4). Therefore, a search for nonhormonal alternatives for contraception is warranted in order to meet the demand for universal access to contraception. Pathological conditions such as luteinized unruptured follicle syndrome, with which women have normal hormonal cycles but do not release mature oocytes and are infertile (5), provide the rationale for novel nonhormonal contraceptives that block ovulation without altering ovarian hormone synthesis and secretion. Several attempts have been made previously to target ovulation via inhibiting prostaglandin synthesis and indeed identified cyclooxygenase 2 (COX-2) inhibitors as potential nonsteroidal contraceptives for emergency use (6, 7). However, efforts to identify ovulation inhibitors suitable for regular contraceptives have stalled due to the lack of appropriate phenotypic screening platforms and our limited knowledge on ovulation, particularly follicle rupture, a final step for releasing the fertilizable oocytes (8, 9).

Our recent work demonstrated that several aspects of ovulation in *Drosophila* are reminiscent of that in mammals. Like mammalian oocytes, *Drosophila* oocytes are also encapsulated by somatic follicle cells, and matrix metalloproteinase (MMP) activity is required for the degradation of the follicle wall and the release of the mature oocyte into the oviduct (10, 11). In addition, reactive oxygen species, particularly hydrogen peroxide, are indispensable for ovulation in both mice and *Drosophila* (12, 13). Although the source of hydrogen peroxide in mammalian follicles has not been well-identified, it is clear that hydrogen peroxide in *Drosophila* follicle cells is derived from NADPH oxidase (NOX) and superoxide dismutase 3 (SOD3) (13). In *Drosophila*, the entire follicle rupture is induced by octopamine (OA), a monoamine equivalent to norepinephrine (NE) (14). OA binds to OAMB (octopamine receptor in the mushroom body; an α-type adrenergic receptor) (15, 16) in follicle cells to induce Ca^2+^ influx, which activates both MMP2 and NOX (14). Similarly, NE is highly enriched in the follicular fluid of preovulatory follicles (17–19); adrenergic receptors are expressed in granulosa cells (20); and adrenergic stimulation and the rise of intracellular calcium also appear to play an important role in mammalian ovulation (21–23). Furthermore, ecdysteroid hormone is also produced in late mature follicles and is essential for *Drosophila* follicle rupture (24), parallel to the progesterone signaling in mammalian ovulation (25–27). Finally, both NR5A-family nuclear receptors, LRH-1 and SF-1 in mammals and Hr39 and Ftz-f1 in *Drosophila*, play important roles in mammalian and *Drosophila* ovulation (28–31). All these similarities led us to propose that *Drosophila* ovulation could be a useful genetic model to screen compounds that target conserved genetic elements to inhibit follicle rupture.

In this study, we screened 1,172 compounds of a Food and Drug Administration (FDA)–approved drug library from Selleckchem using the ex vivo *Drosophila* follicle rupture assay we recently developed (14, 32). We identified several drugs that could effectively inhibit OA-induced follicle rupture and characterized their molecular actions in the inhibition of follicle rupture. We also identified that dexmedetomidine, an α2-adrenergic receptor agonist, can replace OA to induce follicle rupture in a much more potent manner, consistent with the conservation of adrenergic signaling in *Drosophila* and mammals. Using an in vitro three-dimensional (3D) mouse follicle maturation and ovulation assay (33, 34), we demonstrated that three of the four compounds identified as inhibitors in our *Drosophila* model could effectively inhibit human chorionic gonadotropin (hCG)–induced mouse follicle rupture as well. Furthermore, we tested one of the compounds, chlorpromazine, in a mouse superovulation model and demonstrated that chlorpromazine can significantly inhibit mouse follicle rupture in vivo. Therefore, a combination of *Drosophila* and mouse follicle rupture assays is an efficient way to identify small-molecule ovulation inhibitors that could lead to novel nonsteroidal contraceptive development.

## Results

### Pharmacological Screening of 1,172 FDA-Approved Drugs Using a *Drosophila* Follicle Rupture Assay.

**Fig. 1.**
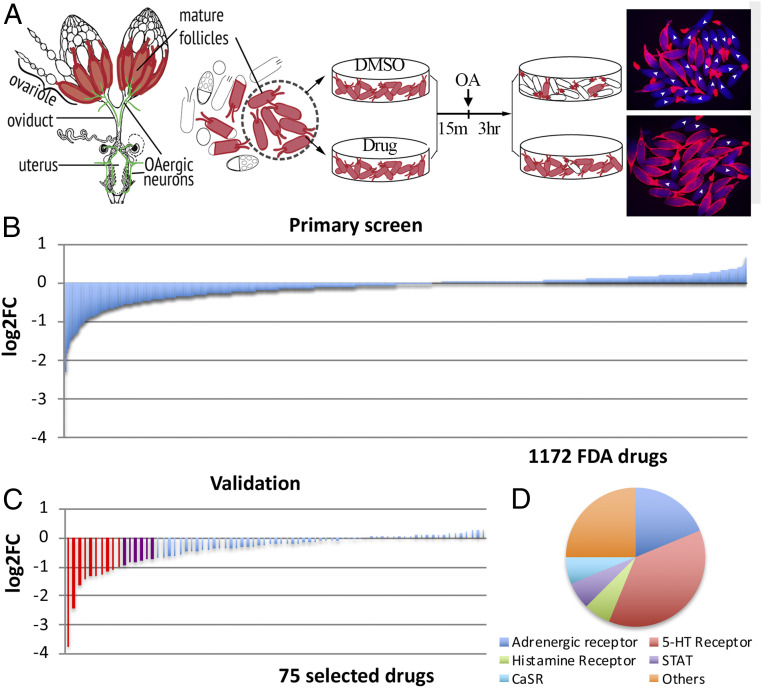
Pharmacological screening of FDA-approved drugs using a *Drosophila* follicle rupture assay. (*A*) A schematic of the drug screening using the ex vivo follicle rupture assay. Mature follicles with an intact follicle-cell layer (marked red) were isolated from *Drosophila* ovaries and pretreated with DMSO or drugs for 15 min before adding OA at 20 μM for 3 h. (*A*, *Right*) Representative images showing follicles after a 3-h OA culture. Ruptured follicles are marked with white arrowheads. (*B*) The primary screen result of 1,172 FDA-approved drugs. The *y* axis is the log2-transformed fold change of rupture rates between the individual drug groups and DMSO groups. (*C*) The validation result of 75 selected drugs identified from the primary screen. The red bars are drugs with log2FC <−1 and the purple bars are drugs with −1< log2FC <−0.75. (*D*) The categories of 16 identified candidate drugs. Also see *SI Appendix*, Table S1.

The similarities of *Drosophila* and mammalian ovulation led us to propose that compounds inhibiting *Drosophila* ovulation have high potential to inhibit mammalian ovulation and be developed into nonsteroidal contraceptives. To prove this concept, we used our ex vivo *Drosophila* follicle rupture assay to identify compounds that could inhibit OA-induced follicle rupture. We selected an FDA-approved drug library from Selleckchem, which contains 1,172 FDA-approved drugs that target a variety of molecules including G protein–coupled receptors (GPCRs), non-GPCR membrane receptors, cytosolic and nuclear receptors, and various enzymes, including kinases. The drug-screening workflow consisted of a primary screening of singular drug groups and a validation step with triplicate drug groups. Isolated mature follicles were pretreated with either the tested drugs at 10 μM or vehicle (DMSO; dimethyl sulfoxide) for 15 min and then stimulated by an addition of 20 μM OA to the media to induce follicle rupture for 3 h (Fig. 1*A*). The fold change (FC) of follicle rupture rate for each drug was then calculated and log2-transformed (log2FC), which makes it easier to visualize the inhibitory vs. stimulatory effects.

From the initial screening, 36 out of 1,172 tested drugs (3.1%) showed log2FC <−1 (more than twofold reduction) and were subjected to validation. To prevent missing potential candidates, we also included another 39 drugs that were close to the cutoff of log2FC = −1. Among these 75 drugs selected for validation, 10 drugs consistently showed log2FC <−1, and 6 drugs showed log2FC between −0.75 and −1 (Fig. 1*C* and *SI Appendix*, Table S1). Niclosamide, a medication used for tapeworm infection (34, 35), showed the most prominent inhibition of follicle rupture. In addition, a majority of these inhibitory drugs target adrenergic, serotonin, dopamine, and histamine receptors.

### Six out of Eight Candidate Drugs Are Confirmed to Inhibit OA-Induced Follicle Rupture in *Drosophila*.

**Fig. 2.**
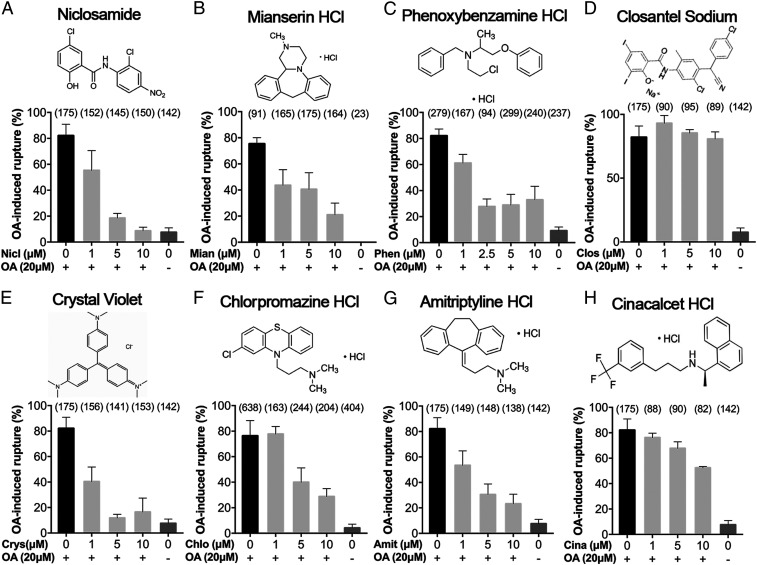
Dose–response analysis of eight inhibitory drugs. Niclosamide (*A*), mianserin (*B*), phenoxybenzamine (*C*), crystal violet (*E*), chlorpromazine (*F*), and amitriptyline (*G*) showed dose-dependent inhibition of OA-induced follicle rupture. In contrast, closantel (*D*) and cinacalcet (*H*) did not show efficient inhibition of OA-induced follicle rupture. A “0” means DMSO was added as vehicle control. The number of follicles used is in parentheses. The 2D structure of each drug is showed under its name.

To further validate the inhibitory effect of these candidate drugs on OA-induced follicle rupture, we randomly selected eight candidates and purchased fresh batches of dry compound to determine their dose–response curve on OA-induced follicle rupture (Fig. 2). The inhibitory effects of all selected candidates, except two (closantel and cinacalcet), could be validated (Fig. 2 and *SI Appendix*, Fig. S1). Closantel and cinacalcet showed no or reduced inhibition in comparison with previous screen results (Fig. 2 *D* and *H*) and are false positives. This discrepancy could have resulted from the breakdown of screening compounds due to long-term storage. Overall, we validated six out of eight (75%) candidate drugs that are effective in inhibiting OA-induced follicle rupture.

All six drugs showed prominent dose–response effects. Niclosamide, one of the essential medicines on the World Health Organization’s list, is the most potent, with a half-maximal inhibitory concentration around 1.2 μM in inhibiting OA-induced follicle rupture (Fig. 2*A*). Recent studies have indicated that niclosamide not only is an effective oral antihelminthic drug but also has broad clinical applications for treating cancer, metabolic diseases, and endometriosis (36). Mianserin (Fig. 2*B*) is a tetracyclic antidepressant and exerts its effects via antagonizing histamine, serotonin, and adrenergic receptors (37). Mianserin is closely related to mirtazapine, which is also one of the potential candidates from our screening and less potent than mianserin (*SI Appendix*, Table S1). Phenoxybenzamine (Fig. 2*C*) was used as an antihypertensive agent due to its irreversible antagonistic effect on α-adrenergic receptors and was an investigational drug for male contraception (38, 39). Chlorpromazine (Fig. 2*F*) is an antipsychotic medication used to treat schizophrenia and its mechanism of action is not entirely clear but believed to be related to its antagonistic effect on dopamine, serotonin, and histamine. In treating schizophrenia, chlorpromazine is as effective as and less effective than asenapine and aripiprazole, respectively (40). Interestingly, both asenapine and aripiprazole are also candidate hits from our screening (*SI Appendix*, Table S1). Amitriptyline (Fig. 2*G*) is also a tetracyclic antidepressant like mianserin and acts by blocking the reuptake of both serotonin and norepinephrine (41). It could also inhibit serotonin, histamine, and adrenergic receptors. Crystal violet (Fig. 2*E*) is a triarylmethane dye for topical antiseptics and may cause cell death; therefore, it was excluded from further investigation. Together, we identified six FDA-approved drugs that can inhibit OA-induced follicle rupture in *Drosophila*.

### Dexmedetomidine Is Identified as a Potent OAMB Agonist.

**Fig. 3.**
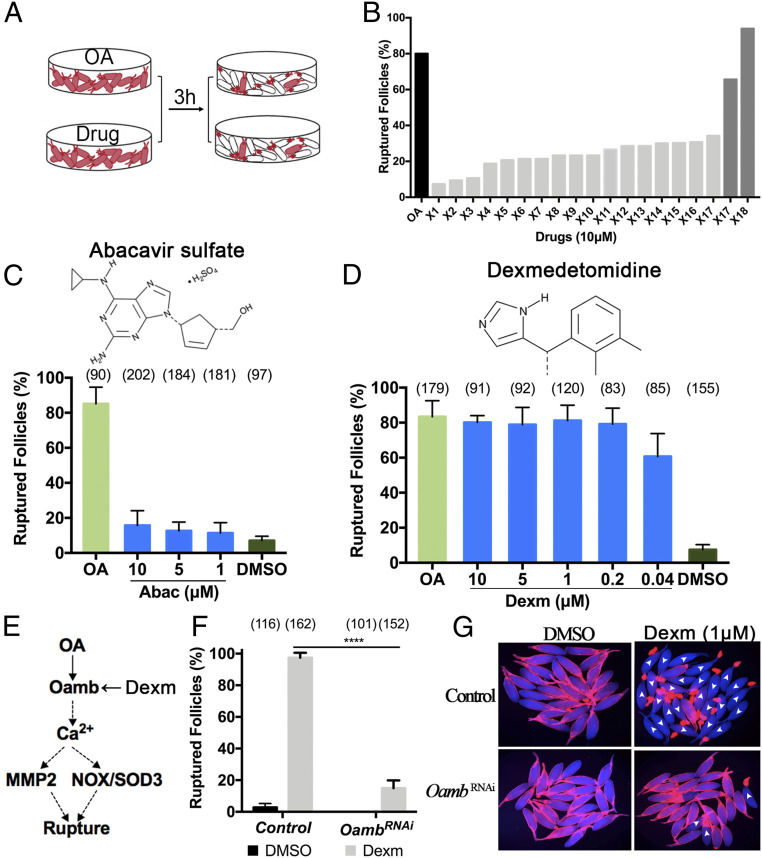
Dexmedetomidine is a potent agonist of the OAMB receptor. (*A*) The screening schematic for drugs that induce follicle rupture without OA. (*B*) The rupture result of 18 potential stimulatory drugs from the primary screening. The black bar is a positive control of OA at 20 μM. (*C* and *D*) The dose–response analysis of abacavir (*C*) and dexmedetomidine (*D*) in inducing follicle rupture. The number of follicles used is in parentheses. (*E*) A schematic of the major signaling pathways involved in *Drosophila* follicle rupture. Dexmedetomidine is an OAMB agonist. (*F* and *G*) Dexmedetomidine-induced follicle rupture is blocked by *Oamb* knockdown in follicle cells. (*F*) The quantification of dexmedetomidine-induced follicle rupture rate using control and *Oamb*-knockdown mature follicles. ****P < 0.0001. (*G*) Representative images of mature follicles after dexmedetomidine (1 μM) treatment for 3 h. The ruptured follicles are marked with white arrowheads. All error bars are SD unless otherwise noted.

Agonists that could induce follicle rupture without OA, such as ionomycin (42), are extremely valuable to decipher the molecular mechanisms of genetic elements and inhibitory drugs (see below). From our primary screening, we also found that 19 drugs (1.6%) enhanced OA-induced follicle rupture to ∼100% in comparison with ∼80% in the DMSO group (log2FC > 0.26; Fig. 1*B*). Some of these drugs may facilitate OA/OAMB signaling to enhance follicle rupture or function as an agonist to induce follicle rupture in the absence of OA. To determine whether any of these drugs can induce follicle rupture in the absence of OA, we treated mature follicles with each drug (10 μM) for 3 h and examined their rupture rate (Fig. 3*A*). Two of the tested drugs, abacavir and dexmedetomidine, showed promising induction of follicle rupture, similar to OA (Fig. 3*B*). We then purchased fresh batches of the dry compound and determined their dose–response. Unfortunately, the newly purchased abacavir did not show efficient induction of follicle rupture even at 10 μM (Fig. 3*C*). In contrast, dexmedetomidine consistently induced maximal follicle rupture at a concentration as low as 0.2 μM (Fig. 3*D*), much more potent than OA, which induces the maximal follicle rupture at 20 μM (14).

Dexmedetomidine is an agonist of the α2-adrenergic receptor, which is homologous to OAMB in *Drosophila*. This led us to hypothesize that dexmedetomidine activates OAMB to induce follicle rupture (Fig. 3*E*). Consistent with this idea, dexmedetomidine-induced follicle rupture was blocked when *Oamb* was knocked down in mature follicle cells using RNA interference (RNAi; Fig. 3 *F* and *G*). Therefore, dexmedetomidine also functions as an OAMB agonist, suggesting the high similarity of OA/OAMB to the mammalian adrenergic system.

### Mechanisms of Action of Ovulation Inhibitory Drugs in *Drosophila*.

**Fig. 4.**
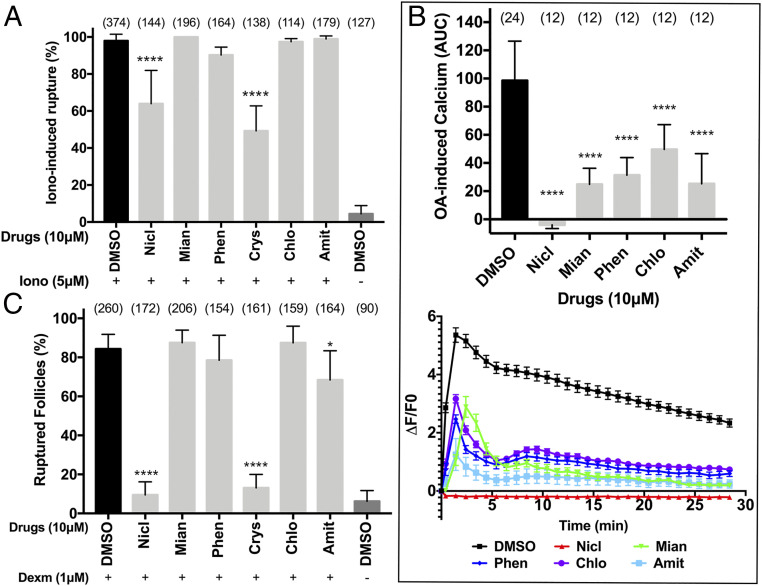
Identification of the mechanisms of action for inhibitory drugs using ionomycin and dexmedetomidine. (*A*) Quantification of ionomycin-induced follicle rupture. Mature follicles were pretreated with drugs (at 10 μM) or DMSO for 15 min before adding ionomycin (at 5 μM) for 3 h. The number of follicles is listed in parentheses. (*B*) Quantification of the OA-induced Ca^2+^ influx using *GCaMP6f*. The AUC (*Upper*; mean *± SD*) and the trace of the calcium signal (*Lower*; Δ*F*/*F*0; mean ± SEM) within 30 min of 20 μM OA stimulation. The number of replicates is shown in parentheses. (*C*) Quantification of dexmedetomidine-induced follicle rupture. The number of follicles used is in parentheses. *P < 0.05 and ****P < 0.0001 when compared with DMSO control. Amit, amitriptyline; Chlo, chlorpromazine; Crys, crystal violet; Mian, mianserin; Nicl, niclosamide; Phen, phenoxybenzamine. All error bars are SD unless otherwise noted.

Next, we set out to investigate how the ovulation inhibitory drugs we identified from our screening block OA-induced follicle rupture. OA activates the OAMB receptor in follicle cells to induce Ca^2+^ influx, which leads to follicle rupture through activation of MMP2 and NOX (Fig. 3*E*) (11, 13, 14). Ionomycin, a potent Ca^2+^ ionophore, can bypass OA/OAMB to induce Ca^2+^ influx and follicle rupture (14). To determine whether these drugs target components upstream or downstream of Ca^2+^ influx, we tested whether these drugs can inhibit follicle rupture induced by ionomycin. Mianserin, phenoxybenzamine, chlorpromazine, and amitriptyline were not able to inhibit ionomycin-induced follicle rupture, suggesting that they target components upstream of Ca^2+^ influx (Fig. 4*A*). Consistent with this result, all four drugs suppressed OA-induced Ca^2+^ influx (Fig. 4*B*). The fact that all four drugs are GPCR antagonists led us to propose that they are OAMB antagonists as well. To test this hypothesis, we examined whether these drugs can inhibit dexmedetomidine-induced follicle rupture. Since dexmedetomidine is much more potent than OA, these drugs may not be able to compete with dexmedetomidine to bind to the OAMB receptor and thus will not be able to inhibit follicle rupture. Consistent with this prediction, all four drugs were not effective in inhibiting dexmedetomidine-induced follicle rupture (Fig. 4*C*; compare with Fig. 2). Therefore, our data suggest that mianserin, phenoxybenzamine, chlorpromazine, and amitriptyline are OAMB antagonists that inhibit OA-induced follicle rupture.

In contrast to those four drugs, niclosamide was able to inhibit ionomycin-induced follicle rupture, although less effectively (Fig. 4*A*). In addition, niclosamide suppressed OA-induced Ca^2+^ influx and effectively inhibited dexmedetomidine-induced follicle rupture (Fig. 4 *B* and *C*). These data suggest that niclosamide may target components both upstream and downstream of Ca^2+^ influx to inhibit follicle rupture. Alternatively, niclosamide could inhibit other pathways involved in follicle rupture or affect the viability of the follicles.

### Three out of Four Drugs Showed Inhibition of Mouse Follicle Rupture.

**Fig. 5.**
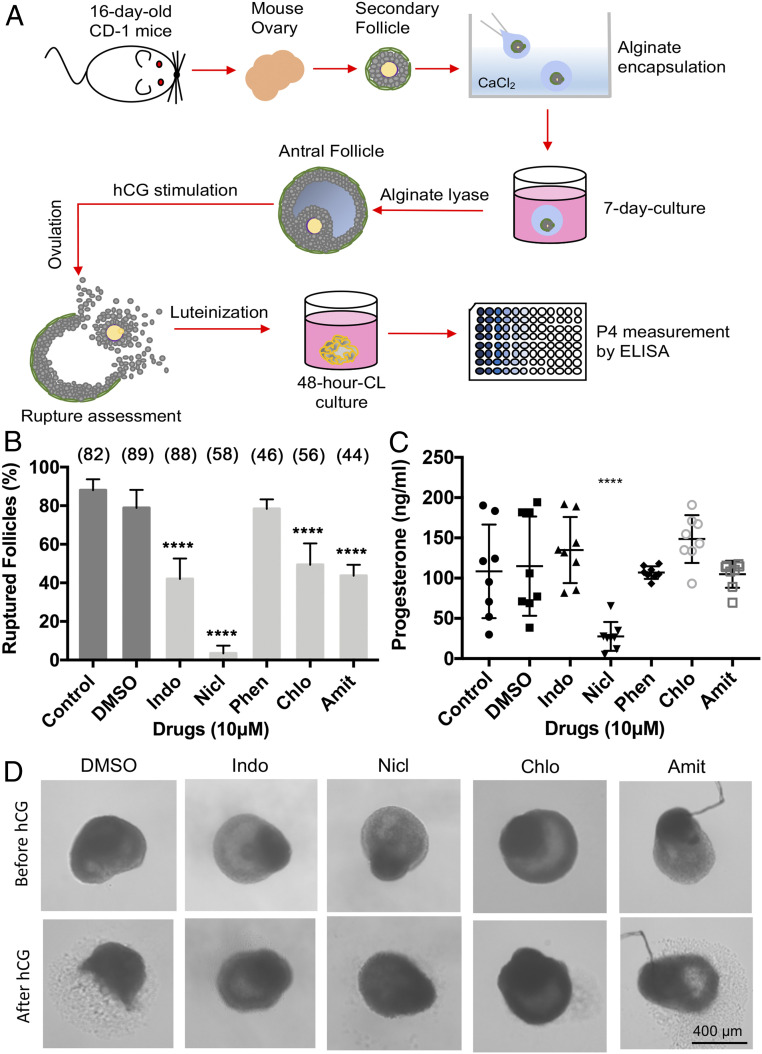
Three out of the four drugs showed inhibition of mouse follicle rupture. (*A*) A schematic of mouse in vitro follicle maturation and the hCG-induced follicle rupture assay. (*B*) The effect of candidate drugs on hCG-induced mouse follicle rupture. ****P < 0.0001 between control and drug treatment groups. The number of follicles is listed in parentheses. (*C*) The influence of candidate drugs on hCG-induced progesterone production. ****P < 0.0001 between the control and Nicl group. (*D*) Representative images of mouse follicles treated with different drugs show successful or failed follicle rupture upon hCG stimulation.

We next asked whether these inhibitory drugs can inhibit follicle rupture in mammals. To address this question, we utilized an in vitro mouse follicle maturation and ovulation assay in a 3D follicle-culture system (Fig. 5*A*) (43). In this system, mouse secondary follicles were isolated, cultured in vitro to maturity, and induced to rupture with hCG stimulation (Fig. 5*A*). More than 80% of follicles were ruptured after hCG stimulation, and the addition of DMSO to the culture medium during hCG stimulation did not affect the rupture rate (Fig. 5*B*). In contrast, the addition of indomethacin, one of the known COX inhibitors that inhibit ovulation in mice and rats (44–46), to culture medium at 10 μM concentration caused a twofold reduction in rupture rate (Fig. 5*B*). Excitingly, three of the four drugs (niclosamide, chlorpromazine, and amitriptyline) at 10 μM showed similar inhibition of mouse follicle rupture, while the other one, phenoxybenzamine, did not show significant inhibition (Fig. 5 *B* and *D*). In addition, all three drugs, niclosamide, chlorpromazine, and amitriptyline, showed a dose-dependent inhibition of follicle rupture (*SI Appendix*, Fig. S2*A*).

To evaluate whether these drugs affect hCG-induced progesterone production, which is a hallmark of luteinization, we continued to culture formed corpus luteum (CL) for 48 h and collected conditioned media for progesterone measurement. Our results showed that there were comparable levels of progesterone secretion between control, DMSO, indomethacin, phenoxybenzamine, chlorpromazine, and amitriptyline treatment groups (Fig. 5*C* and *SI Appendix*, Fig. S2*B*). This suggests that indomethacin, chlorpromazine, and amitriptyline only affect hCG-induced follicle rupture but not luteinization. In contrast, the progesterone level was significantly reduced in the niclosamide treatment group (Fig. 5*C*), suggesting that niclosamide not only affects hCG-induced follicle rupture but also luteinization. Alternatively, niclosamide may lead to cytotoxicity.

### Chlorpromazine Can Significantly Inhibit hCG-Induced Mouse Follicle Rupture in Vivo.

**Fig. 6.**
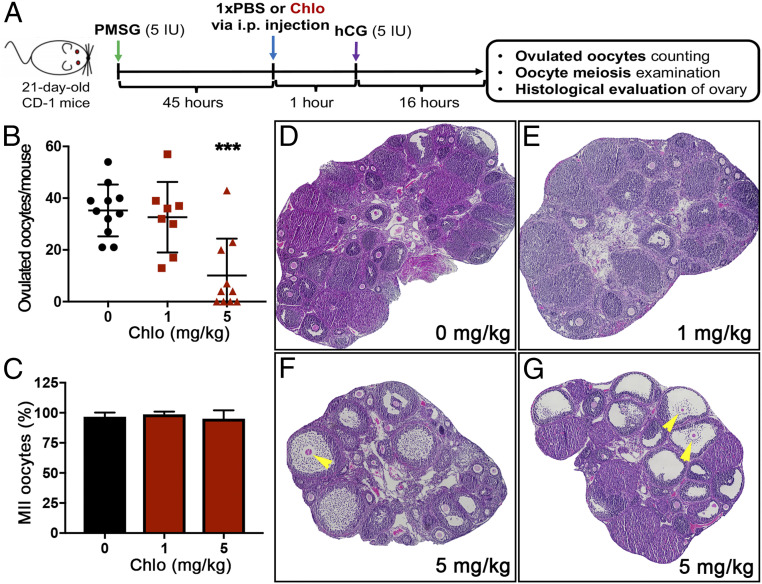
Chlorpromazine can significantly inhibit mouse follicle rupture in vivo. (*A*) A schematic shows the procedure of the mouse superovulation model. (*B*) Quantification of ovulated oocytes from mice treated with chlorpromazine at 0, 1, and 5 mg/kg at 1 h before hCG treatment. ***P < 0.001 compared with the control group; *n* = 8 to 11 female mice in each treatment group. (*C*) Quantification of MII oocytes for the ovulated oocytes in the oviduct of mice treated with chlorpromazine at 0, 1, and 5 mg/kg at 1 h before hCG treatment. (*D*–*G*) Representative histological images of ovaries at 16 h after hCG treatment in mice treated with chlorpromazine at 0 (*D*), 1 (*E*), and 5 (*F* and *G*) mg/kg. Unruptured follicles with entrapped oocytes are marked by yellow arrowheads.

Chlorpromazine has been shown to inhibit ovulation through affecting the hypothalamus–pituitary axis and its associated luteinizing hormone surge in rats (47, 48). Our in vitro mouse follicle ovulation assay suggests that chlorpromazine also acts locally in follicles to inhibit follicle rupture. We further used an in vivo mouse superovulation model to test whether chlorpromazine can directly target ovaries to inhibit ovulation (Fig. 6*A*). The mice treated with vehicle and 1 mg/kg chlorpromazine through intraperitoneal injection ovulated comparable numbers of oocytes (35.3 ± 10.0 vs. 32.6 ± 13.6, respectively) in the oviduct after hCG treatment (Fig. 6*B*). However, mice treated with 5 mg/kg chlorpromazine had significantly fewer ovulated oocytes (10.1 ± 14.3), with 4 out of 10 mice having no oocytes detected in the oviducts (Fig. 6*B*). For the harvested oocytes in all three groups, there were comparable percentages of metaphase II (MII) oocytes and more than 96.0% of them had the first polar body extrusion (Fig. 6*C*), indicating that chlorpromazine did not affect oocyte meiosis if they were ovulated.

The ovarian histological staining results further revealed that ovaries from vehicle and 1 mg/kg chlorpromazine-treated mice had the expected presence of CL and absence of preovulatory follicles (Fig. 6 *D* and *E*). However, in the ovaries treated with 5 mg/kg chlorpromazine, there were markedly more unruptured follicles that had oocytes trapped inside; in addition, these unruptured follicles had undergone cumulus expansion (Fig. 6 *F* and *G*). This ovarian phenotype is reminiscent of ovaries with granulosa cell–specific deletion of progesterone receptor (PR), an essential nuclear receptor to regulate proteolysis and extracellular matrix (ECM) remodeling during follicle rupture (25, 49, 50). Taken together, these results demonstrate that chlorpromazine can locally inhibit mouse ovulation in vivo by blocking the rupture of preovulatory follicles. In conclusion, our work showed that compounds inhibiting *Drosophila* follicle rupture have the potential to inhibit mammalian ovulation without interfering in hormone production and that *Drosophila* ovulation could be a useful platform to screen nonsteroidal contraceptive compounds.

## Discussion

An inexpensive and robust phenotypic screening platform is very valuable for nonsteroidal contraceptive development and to meet the demand of current contraceptive needs. With our previous genetic studies showing similarity between *Drosophila* and mammalian ovulation, we thus proposed to develop *Drosophila* ovulation as a screening platform to profile compound properties in inhibition of ovulation, an essential step in reproduction and a validated strategy for developing nonsteroidal contraceptives (6, 7). Supporting this concept, *Drosophila* has been successfully used in several anticancer drug screenings (51–53). As a proof of principle, this study screened 1,172 FDA-approved drugs and identified six drugs that could inhibit *Drosophila* follicle rupture in a dose-dependent manner. We demonstrated that three out of the four drugs could also inhibit hCG-induced mouse follicle rupture in vitro and that two of them did not affect progesterone production and luteinization. Most strikingly, we also demonstrated that chlorpromazine is able to inhibit hCG-induced follicle rupture and ovulation in vivo. Overall, our work suggests that *Drosophila* follicle rupture, in combination with mouse follicle rupture, is a valuable screening platform for identification of lead compounds for nonsteroidal contraceptive development. The utilization of two genetic models (*Drosophila* and mice) may be more advantageous to identify lead compounds that will work in humans than the single model. On the other hand, compounds that only affect *Drosophila* ovulation but not mouse ovulation could lead to developing safer chemicals for insect population control.

*Drosophila* is not only inexpensive for chemical screens but also has a wealth of genetic tools for target deconvolution and identification of lead compounds. Using genetic tools, we were able to pinpoint that mianserin, phenoxybenzamine, chlorpromazine, and amitriptyline inhibit OA-induced follicle rupture by antagonizing the OAMB receptor (Fig. 4). We also demonstrated that dexmedetomidine, an α2-adrenergic agonist, acts on the OAMB receptor to induce follicle rupture. These data further support the notion that the *Drosophila* octopamine system is equivalent to the mammalian adrenergic system (54). The essential role of OA in *Drosophila* follicle rupture and the inhibition of mouse follicle rupture by chlorpromazine and amitriptyline lead us to propose that the adrenergic system plays a much more conserved role in follicle rupture across multiple species.

Among the three candidate drugs identified, chlorpromazine and amitriptyline are promising compounds for further investigation, since both of them inhibit mouse follicle rupture in vitro and do not affect progesterone production. Chlorpromazine has long been known to inhibit ovulation through regulating the hypothalamus–pituitary axis (47, 48, 55–57). Our work here suggests that chlorpromazine could also directly act on mature follicles to inhibit follicle rupture in vivo. The apparent difference between our work and previous reports could be due to the difference of dose used, site and time of injection, and/or species. However, whether chlorpromazine antagonizes adrenergic receptors in mammalian follicles as in *Drosophila* follicles awaits further investigation. Alternatively, chlorpromazine may influence adrenergic signaling by inhibiting clathrin-coated vesicle formation and thus endocytosis of membrane receptors (58, 59). The similarity of the ovarian phenotype between chlorpromazine-treated and PR-knockout mice also leads us to propose that chlorpromazine might target PR-associated proteolysis and ECM remodeling in follicular granulosa cells during follicle rupture. Amitriptyline, an antidepressant drug for migraines, has not been well-studied in the reproductive process and is not linked to defective ovulation (60). In contrast, amitriptyline seems to improve ovary morphology and functions in rats with estradiol valerate–induced polycystic ovary by modulating ovarian adrenergic signaling (61). In that sense, it is plausible that amitriptyline may also regulate adrenergic signaling during hCG-induced follicle rupture. In line with this prediction, multiple adrenergic receptor regulators can influence ovulation in rabbits, rats, hens, and fish (62–65). All these studies indicate the necessity to decipher the role of follicular adrenergic signaling in follicle rupture and ovulation in mammals, including humans.

Although niclosamide is the most potent in inhibiting follicle rupture in both *Drosophila* and mice, it also inhibits progesterone production after hCG stimulation. It is uncertain whether niclosamide’s inhibitory effect on follicle rupture is due to inhibition of ovulatory signaling or its cytotoxicity. However, it is clear that niclosamide does not cause acute cell toxicity in *Drosophila* follicles. We performed trypan blue staining of niclosamide-treated follicles and only observed minimal, if any, follicle cell death (*SI Appendix*, Fig. S3). This is further confirmed by cleaved caspase 3 antibody staining (*SI Appendix*, Fig. S4), which was used to recognize apoptotic follicle cells (66). Despite these results, we cannot exclude the possibility that the chronic toxicity of niclosamide contributes to its effect on mouse follicle rupture inhibition and progesterone secretion. Recent work has reported that niclosamide can disrupt multiple signaling pathways, including NFκB, STAT3, and WNT signaling, in a variety of cancer models and has broad clinical implications (36). In addition, niclosamide is in a phase II clinical trial for treating COVID-19 infection. Furthermore, niclosamide also showed a therapeutic effect on endometriosis in a mouse model (67), in which researchers did not find any reproductive defect after niclosamide treatment. This apparent difference from our result could be due to the low serum availability of niclosamide in the previous study. It is worth noting that niclosamide through vaginal implantation is being considered for nonsteroidal female contraceptives by preventing sperm migration through the female reproductive tract via draining of the sperm cell’s energy (http://mcb.berkeley.edu/news-and-events/transcript/fall-2019-revolution-contraception). Therefore, future work will be required to identify the potential targets of niclosamide in ovulation inhibition.

## Materials and Methods

### Chemicals and the FDA-Approved Drug Library.

A library of 1,172 FDA-approved drugs was obtained from Selleckchem (Z71924) and stored in a −80 °C freezer. All drugs were predissolved in DMSO as 10 mM stock solutions. The following chemicals were also used: DMSO (Sigma; 276855), octopamine (Sigma; O0250), ionomycin (Cayman Chemical; 11932), niclosamide (Selleckchem; S3030), mianserin hydrochloride (Selleckchem; S1382), phenoxybenzamine hydrochloride (Sigma; B019), closantel sodium (Selleckchem; S4105), crystal violet (Selleckchem; S1917), chlorpromazine hydrochloride (Sigma; C8138), amitriptyline hydrochloride (Selleckchem; S3183), cinacalcet hydrochloride (Selleckchem; S1260), abacavir sulfate (Selleckchem; S3165), dexmedetomidine (Selleckchem; S2090), and indomethacin (Cayman Chemical; 70270).

### *Drosophila* Genetics.

Flies were reared on standard cornmeal-molasses food at 25 °C, unless noted otherwise. *47A04-Gal4*, the Gal4 line expressed in mature (stage 14) follicle cells (14), was used in all the genetic crosses for isolating mature follicles. *UAS-RG6*, which contains a *RanGAP::mCherry* fusion gene under the control of the upstream activation sequence (UAS) (68), was used to report Gal4 expression. To produce flies for the ex vivo follicle rupture assay, virgin females *UAS-dcr2; 47A04-Gal4, UAS-RG6* were crossed to wild-type male *Oregon-R* or male *UAS-Oamb*^*RNAi*^ (V2861 from the Vienna *Drosophila* Resource Center) for knocking down *Oamb* genes in mature follicle cells. Animals were shifted to 29 °C in late pupal and adult stages to increase Gal4 expression level. For measuring Ca^2+^ influx, virgin females *47A04-lexA, lexAop-GCaMP6f; Oamb-RFP[M2]* were used to cross to *Oregon-R* males. *Oamb-RFP* is a reporter expressed in mature follicle cells (69).

### Ex Vivo Follicle Rupture Assay and Screening Procedures.

The ex vivo follicle rupture assay was similar to one previously described with slight modifications (32). In short, 6-d-old virgin female flies fed wet yeast for 3 d were used to isolate mature follicles in Grace’s insect medium (Caisson Labs). Within an hour, isolated mature follicles were distributed into groups of ∼30 follicles and cultured with 1 mL of culture medium (Grace’s medium + 10% fetal bovine serum [FBS] + 1× penicillin/streptomycin). All cultures were carried out in a 29 °C incubator. For inhibitory compound screening, individual drugs or DMSO control were added to the culture medium 15 min before adding OA (20 μM), ionomycin (5 μM), or dexmedetomidine (1 μM). Three hours after adding ovulatory stimuli, mature follicles were imaged using a Leica MZ10F fluorescence stereoscope with a sCOMS camera (PCO.Edge), and the number of ruptured follicles was counted manually. One data point represents the percentage of ruptured follicles per experimental group (∼30 follicles). The fold change of rupture rate for each drug is calculated by the rupture rate of the drug group divided by the rupture rate of the DMSO group.

### Measurement of Ca^2+^ Influx.

For measuring the OA-induced rise of intracellular Ca^2+^, mature follicles with GCaMP6f expression in follicle cells were isolated from 6-d-old virgin females and distributed in groups of 15 follicles (10 μL culture medium) into each well of 96-well glass-bottom microplates (Corning; 30621-096), which contained 80 μL Grace’s medium + 1 μL drug (10 mM stock) or DMSO control. Wells with an addition of 10 μL culture medium were used for background normalization. Plates were mixed and cultured for 30 min in a 29 °C incubator before placing into the plate reader (CLARIOstar microplate reader; BMG Labtech) for OA injection (20 μM at the final concentration) and fluorescence reading. The GCaMP6f signal was detected using the following program: 470 ± 15 nm excitation and 515 ± 20 nm emission filters; spiral average, diameter 4 for bottom scan; dynamic reading with 60 s per cycle, 30 cycles. One well represents one data point; Δ*F*/*F*0 was calculated and is plotted in Fig. 4*B*; the area under the curve (AUC) of the Δ*F*/*F*0 trace for each well was calculated using the trapezoid method in Microsoft Excel.

### Animals.

CD-1 mice were housed in polypropylene cages and provided food and water ad libitum. All mice were kept under a temperature-, humidity-, and light- (14 h light/10 h dark) controlled barrier facility. Animals were fed Teklad Global irradiated 2919 or 2916 chow, which does not contain soybean or alfalfa meal to minimize the exposure to phytoestrogens. All animal procedures used in this study were approved by the Institutional Animal Care and Use Committees at Northwestern University and Rutgers University and correspond to the guidelines of the NIH.

### Mouse Follicle Isolation, Encapsulation, and Culture.

Mouse follicle isolation, encapsulation, and culture were carried out as previously described (33, 34, 70, 71). In short, morphologically normal multilayered secondary follicles (150 to 180 μm in diameter) were mechanically isolated and selected from 16-d-old CD-1 female mice in L15 media (Invitrogen) containing 1% FBS (Invitrogen). Follicles were then incubated in the maintenance media (50% minimal essential medium [αMEM GlutaMAX; Invitrogen] and 50% nutrient mixture [F-12 with GlutaMAX]) with 1% FBS at 37 °C, 5% CO_2_ in air for 2 h. Afterward, individual follicles were encapsulated in 5 μL 0.5% (weight/volume) alginate hydrogel (Sigma-Aldrich), immediately immersed in 50 mM CaCl_2_ and 140 mM NaCl for 2 min to allow cross-linking, and incubated in the maintenance media to recover for 2 h. Individual follicles were then cultured in 96-well plates for 7 d in the follicle-culture media (maintenance media + 3 mg/mL bovine serum albumin + 10 mIU/mL human recombinant follicle-stimulating hormone [FSH; from A. F. Parlow, National Hormone and Peptide Program, National Institute of Diabetes and Digestive and Kidney Diseases, Bethesda, MD] + 1 mg/mL bovine fetuin [Sigma-Aldrich] + ITS [5 μg/mL insulin, 5 μg/mL transferrin, and 5 ng/mL selenium; Sigma-Aldrich]). Half of the follicle-culture media was replaced every other day.

### In Vitro Ovulation, Compound Exposure, and Follicle Rupture Assessment.

In vitro ovulation was performed after 7 d of follicle culture as previously described (34). Grown antral follicles were removed from alginate beads by incubating alginate beads in L15 media containing 1% FBS and 10 IU/mL alginate lyase from *Flavobacterium multivorum* (Sigma-Aldrich) at 37 °C for 20 min. Follicles were then incubated for 14 h in the ovulation media (αMEM with 10% FBS, 1.5 IU/mL hCG [Sigma-Aldrich], 10 ng/mL epidermal growth factor [BD Biosciences], and 10 mIU/mL FSH) at 37 °C in 5% CO_2_ in air for ovulation induction. For compound-treated groups, DMSO or drugs were added to the ovulation media to obtain a final concentration of 10 μM. After a 14-h incubation, follicles were imaged using the EVOS FL Auto Imaging System with 10× objectives (Thermo Fisher Scientific) to examine follicle rupture. Follicles that broke from one side of the follicular wall and had an expanded cumulus–oocyte complex were defined as “ruptured” follicles, and follicles with intact follicular wall and antrum were defined as “unruptured” follicles.

### Progesterone Production and Measurement.

Upon in vitro ovulation induction, hCG-treated follicles were washed three times in the follicle-culture media without FSH to remove residue-exposed compounds, and transferred to 96-well plates individually with each well containing 100 μL follicle-culture media without FSH. Follicles were cultured for an additional 48 h at 37 °C in 5% CO_2_ in air. Conditioned media were used to measure progesterone concentrations using enzyme-linked immunosorbent assay (ELISA) kits (PG362S; Calbiotech) according to the manufacturer’s instructions. Media collected from wells without follicles were used as the negative control.

### Mouse Superovulation in Vivo.

Twenty-one-day-old CD-1 female mice were intraperitoneally (IP) injected with 5 IU pregnant mare serum gonadotropin (PMSG; HOR 272; ProSpec) to stimulate early antral follicles to grow to preovulatory stage for maturation, which was followed by another IP injection of 5 IU hCG (C1063; Sigma-Aldrich) at 46 h after PMSG injection to induce ovulation. Chlorpromazine was dissolved in 1× phosphate-buffered saline (PBS) and mice were treated with 0, 1, and 5 mg/kg chlorpromazine via IP injection at 1 h before hCG treatment. Oocytes were harvested from the ampulla region of both sides of the oviduct at 16 h after hCG treatment. Next, the number of ovulated oocytes and the polar body extrusion of ovulated oocytes were examined. The postovulated ovaries were fixed in 10% neutral buffered formalin solution (Thermo Fisher Scientific) for 24 h, embedded in paraffin, and sectioned at a thickness of 5 μm. Eight to 16 ovarian sections were randomly selected and stained with hematoxylin and eosin (Thermo Fisher Scientific) for histological evaluation.

### Statistical Analysis.

All statistical analyses were performed using one-way ANOVA, followed by Dunnett’s multiple-comparisons test where appropriate. Differences were considered significant when *P* ≤ 0.05. All error bars are SD unless otherwise noted.

## Supplementary Material

Supplementary File

## Data Availability

All study data are included in the article and/or *SI Appendix*.
