# Supplementary material for: A platform utilizing Drosophila ovulation for nonhormonal contraceptive screening
Source: Proc Natl Acad Sci U S A. 2021 Jul 6;118(28):e2026403118. doi: 10.1073/pnas.2026403118 (PMC8285897; doi:10.1073/pnas.2026403118)
Supplement: Supplementary File [file pnas.2026403118.sapp.pdf]

PNAS
www.pnas.org

Supplementary Information for

A platform utilizing *Drosophila* ovulation for nonhormonal contraceptive screening

Kewa Jiang[a,2,1,] Jiyang Zhang[b1], Yuping Huang[a1], Yingzheng Wang[c], Shuo Xiao[c], M. Kyle Hadden[d], Teresa K. Woodruff[b3], and Jianjun Sun[a,e,4]

Corresponding authors
Jianjun Sun
Email: jianjun.sun@uconn.edu

This PDF file includes:

Supplementary Materials and Methods
Figures S1 to S4
Table S1
SI References

Supplementary Materials and Methods

Trypan Blue and Caspase 3 antibody staining.

Trypan blue staining, a method for detect membrane-permeable cells, was performed to evaluate the cytotoxicity of candidate drugs. In this assay, mature follicles were isolated in Grace's medium from 6-d-old females with 3 d of wet yeast feeding. Mature follicles were then stained with 4% of trypan blue solution (ThermoFisher,15250061) for 3 min and gently washed with Grace's medium for 3 times. Unstained follicles were then distributed in groups of ~30 into each well with 1 mL culture medium and imaged using the Olympus SZX16 fluorescent stereoscope equipped with an Olympus DP72 color camera. Individual drugs (10μM) or DMSO were then added into each well and cultured for 3 h in a 29 °C incubator. Afterwards, follicles were stained with 4% trypan blue solution again for 3 min, rinsed with Grace's medium for 3 times and imaged with the color camera. Each experiment was repeated 3 times and representative images were shown in Figure S2.

For Caspase 3 antibody staining, about 45 mature follicles were isolated, distributed into each well, and cultured in the culture medium with individual drug for 3.5 h before antibody staining. The staining procedure follows the standard protocol with minor modification (1). In short, mature follicles were fixed in 4% EM-grade paraformaldehyde for 10 minutes, blocked in PBTG (PBS with 0.2% Triton X-100, 0.5% BSA, and 2% normal goat serum), and stained with primary antibody against cleaved Caspase-3 (Asp175; Cell Signaling Technology; 1:100). The Alexa Flour 488 goat secondary antibody (1:1000; Invitrogen) was used. Mature follicles were also stained with 0.1 ug/ml of 4/,6-Diamidino-2-Phenylindole (DAPI) for 10 minutes to label cell nuclei. Images were acquired in a Leica SP8 confocal laser scanning microscope.

Supplemental Figures

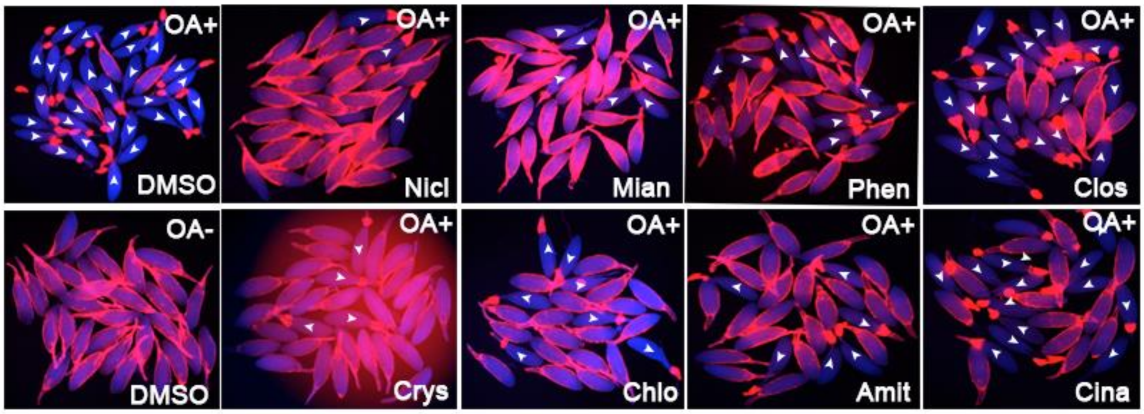

Figure S1. Representative images of mature follicles after 3-h OA stimulation. All panels, except lower left one, were representative images showing mature follicles treated with indicated drugs and 20 μM OA. The lower left panel shows images of follicles without OA treatment. Follicles were imaged with incident light shown in blue and follicle cells are marked by *47A04-Gal4* driving *UAS-RG6* expression in red. Ruptured follicles were marked with arrowheads. Nicl: niclosamide; Mian: mianserin; Phen: phenoxybenzamine; Clos: Closantel; Crys: crystal violet; Chlo: chlorpromazine; Amit: amitriptyline; Cina: Cinacalcet.

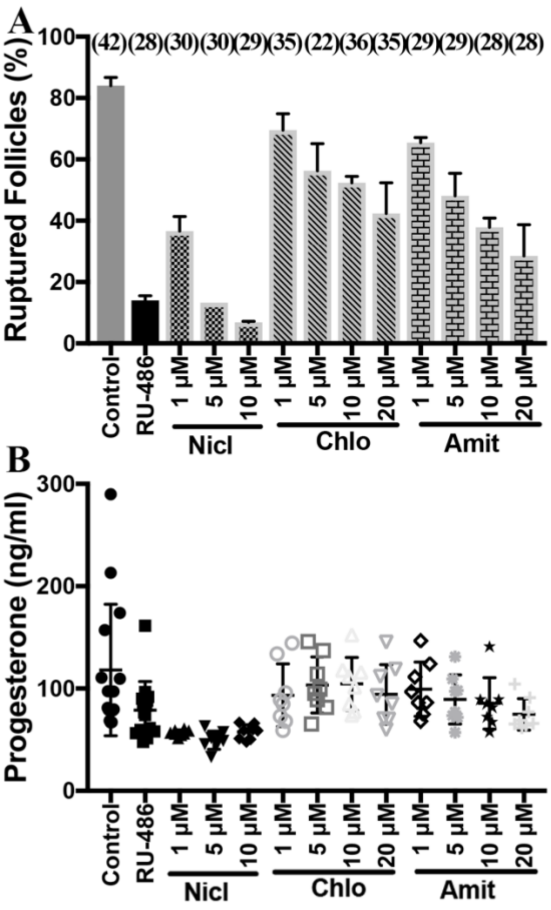

Figure S2. Dose response analysis for candidate drugs in mouse follicle rupture and progesterone production. (A) The dose response of candidate drugs on hCG-induced mouse follicle rupture *in vitro*. Data are plotted as mean ± SD. The number of follicles is listed in brackets. (B) The influence of candidate drugs on progesterone production 48 hours after hCG treatment. RU-486, a potent inhibitor of progesterone receptor, is used at 100 μM as previously reported.

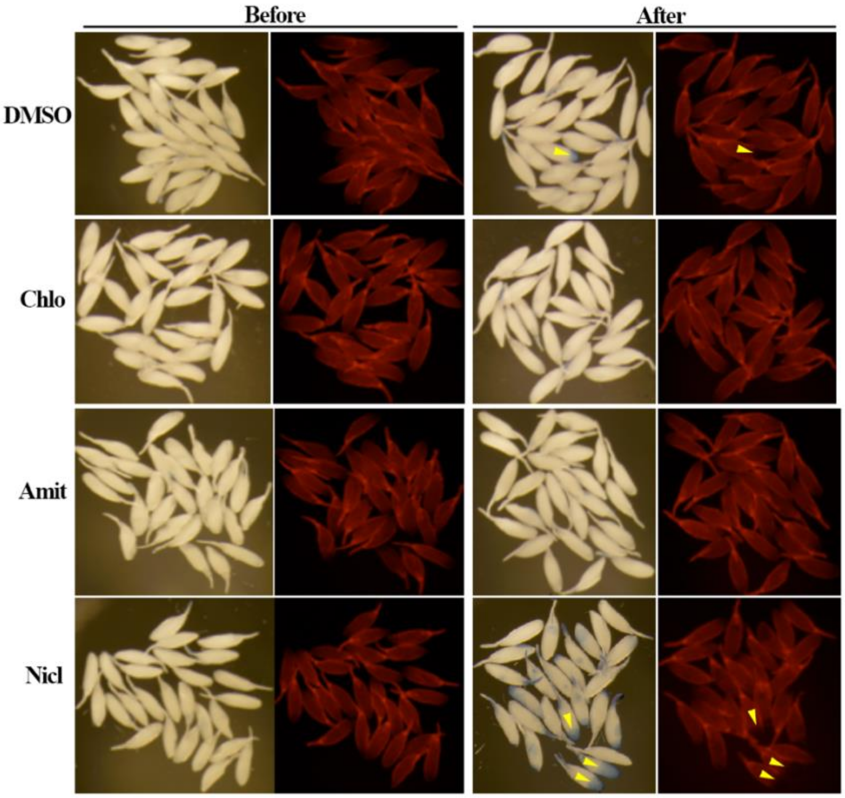

Figure S3. Trypan blue staining of drug-treated mature follicles. Representative images show the trypan blue staining of mature follicles before and after the 3-h treatment with indicated drugs. Follicle cells were marked by *47A04-Gal4* driving *UAS-RG6* expression in red. Note the posterior blue staining corresponding to the lack of red florescent signal (yellow arrowheads), indicating the loss of follicle cells and the staining in underlying oocyte membrane. Chlo: chlorpromazine; Amit: amitriptyline; Nicl: niclosamide.

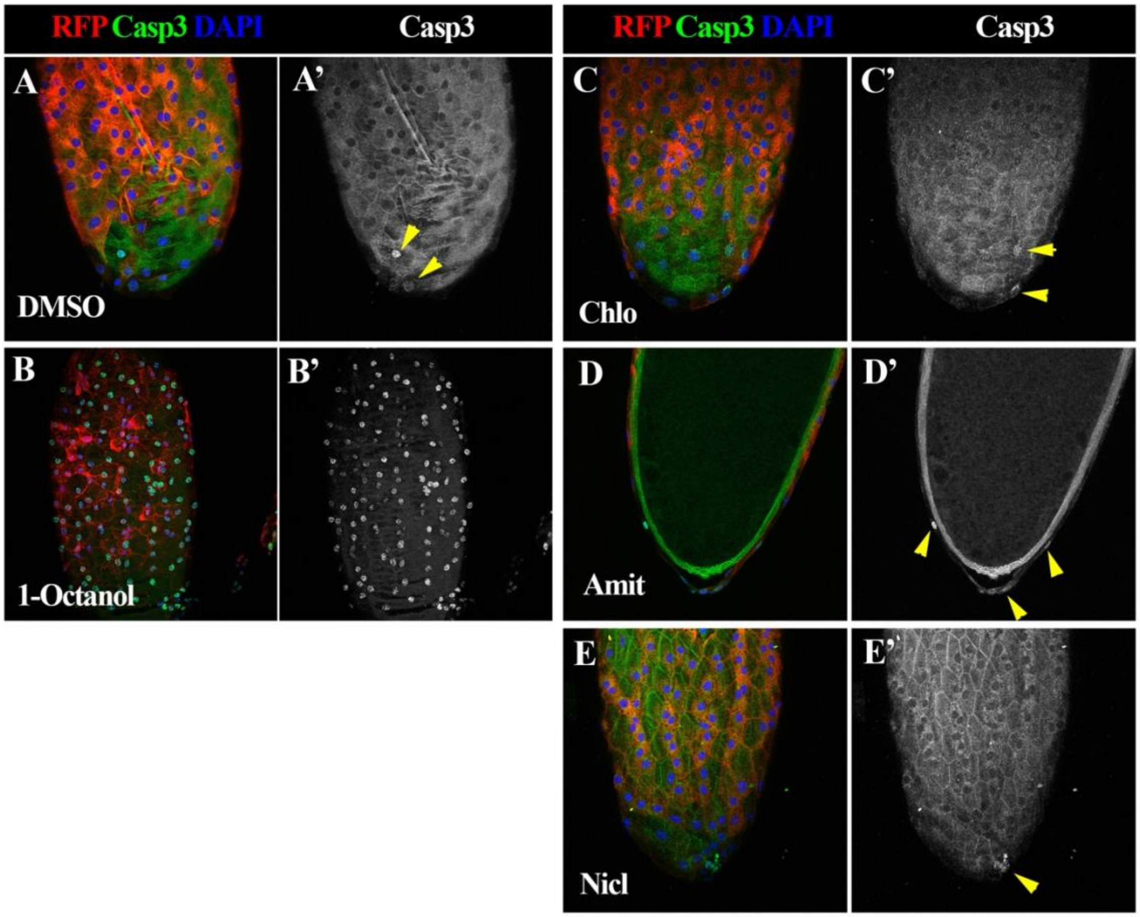

Figure S4. Cleaved Caspase 3 staining of drug-treated mature follicles. Representative images show the cleaved Caspase 3 staining (green in A-E and white in A'-E') of mature follicles treated with DMSO (A), 1-octanol (6 mM; B), chlorpromazine (10 μM; C), amitriptyline (10 μM; D), or niclosamide (10 μM; E). Mature follicle cells are marked by *47A04-Gal4* driving *UAS-RG6* expression (red in A-E) and cell nuclei are labeled with DAPI (blue in A-E). Caspase-3 positive cells were occasionally detected in posterior region of the mature follicles treated with DMSO, chlorpromazine (Chlo), amitriptyline (Amit), and niclosamide (Nicl) for 3.5 h (yellow arrowheads), while all follicle cells were Caspase 3 positive after treatment with 1-octonal for 0.5 h (B and B'). More than 30 follicles were examined in each condition.

Table S1 A list of candidate drugs identified from the validation screening.

#	Drug Name	Catalog (Selleck)	Potential targets	Total follicles	Ruptured follicle (%)	log2FC
1	Niclosamide (Niclocide)	S3030	STAT3	93	5.4	-3.84
2	Mianserin HCl	S1382	Histamine and serotonin receptors	60	13.3	-2.47
3	Phenoxybenzamine HCl	S2499	α-adrenergic receptor	92	29.2	-1.64
4	Closantel Sodium	S4105	the bacterial KinA/Spo0F system	87	28.7	-1.40
5	Crystal violet	S1917	a triarylmethane dye	93	32.3	-1.32
6	Chlorpromazine (Sonazine)	S2456	domapine receptor and potassium channel	90	36.6	-1.32
7	Pizotifen malate	S1394	serotonin receptor	60	30.0	-1.29
8	Amitriptyline HCl	S3183	adrenergic and serotonin receptors	84	34.5	-1.12
9	Asenapine	S1283	adrenergic, serotonin, dopamine, and histamine receptors	96	40.6	-1.09
10	Clozapine (Clozaril)	S2459	serotonin receptor	86	44.2	-1.00
11	Cinacalcet HCl	S1260	CaSR	60	38.3	-0.92
12	Benzthiazide	S4308	others	84	45.2	-0.86
13	Risperidone (Risperdal)	S1615	adrenergic, serotonin, dopamine, and histamine receptors	87	43.7	-0.86
14	Mirtazapine	S2016	adrenergic and serotonin receptors	60	43.3	-0.79
15	Aripiprazole (Abilify)	S1975	serotonin receptor	92	47.8	-0.76
16	Bromocriptine Mesylate		Others	63	46.0	-0.74

SI References

1. E. M. Knapp, W. Li, J. Sun, Downregulation of homeodomain protein Cut is essential for Drosophila follicle maturation and ovulation. *Development* **146** (2019).
